# Flow cytometric characterization of brain dendritic cell subsets after murine stroke

**DOI:** 10.1186/2040-7378-6-11

**Published:** 2014-11-04

**Authors:** Claudia Pösel, Anna Uri, Isabell Schulz, Johannes Boltze, Gesa Weise, Daniel-Christoph Wagner

**Affiliations:** Fraunhofer Institute for Cell Therapy and Immunology, Leipzig, Germany; Translational Centre for Regenerative Medicine, Leipzig, Germany; Department of Neurology, University of Leipzig, Leipzig, Germany

**Keywords:** Cerebral stroke, Brain ischemia, Macrophages, Dendritic cells, Flow cytometry

## Abstract

**Background:**

Sterile inflammation is a substantial element of post-stroke pathophysiology with the determination of autoimmunity versus tolerance being one of its most important aspects. It is believed that this determination is initiated relatively early after stroke onset by clearing macrophages and migratory dendritic cells (DC). However, the phenotypic differentiation of macrophages and DC is intricate particularly in the disease context. Here, we utilized a set of surface markers used in mucosal immunity research to investigate the involvement of macrophages and DC subpopulations in post-stroke inflammation in mice.

**Findings:**

Photothrombotic stroke induced a significant increase of lineage (CD3, B220, Ly6G and CD49b) negative CD11b+ cells in the brain primarily consisting of F4/80+ macrophages and, to a lesser extent, F4/80-/CD11c-/CD11b+ monocytes and F4/80-/CD11c+ DC. The latter could be differentiated into the classical migratory DC subpopulations (CD11b+ and CD103+), but no CD4 or CD8+ DC were found. Finally, stroke caused a significant increase of CD11b/CD103 double-positive DC in the affected brain hemisphere.

**Conclusions:**

The surface marker combination used in this study allowed a phenotypic differentiation of macrophages and DC subpopulations after stroke, thus providing an important prerequisite to study post-stroke immunity and tolerance.

## Background

Ischemic tissue damage during cerebral stroke triggers a prompt and long-lasting immune reaction that involves virtually all parts of the innate and adaptive immune system. The chronology of immune cell infiltration and the interplay of immune cell subsets largely follow the generic course of sterile tissue inflammation.

The mononuclear phagocyte family plays a pivotal role for whether the adaptive immune system responds immunogenic or tolerogenic to ischemic cell death. Macrophages attenuate immunogenic cell death by phagocytosing dying cells and debris, and promote tissue repair [1]. On the other hand, migratory dendritic cells (DC) are capable of transporting antigens towards the draining lymph nodes, and initiate antigen-specific immune responses [2]. After stroke, DC thus shape the adaptive immune reaction to brain antigens either by promoting tolerance or by inducing autoreactive T cells which may finally deteriorate stroke outcome [3].

Though controversially discussed [4], the idea of a “functional labor division” between macrophages and DC was substantiated by the finding that different transcriptional programs are expressed in both populations [5]. However, despite these apparent functional differences, it is still unclear if and how macrophages and DC could be phenotypically differentiated, especially during inflammation [6]. This shortcoming particularly applies for the ischemic brain, where some evidence for DC activity and tolerance induction exists [7, 8], but detailed mechanistic studies are currently hampered by a lack of specific DC markers.

Studies on the role of DC during post-stroke inflammation have been primarily based on the use of the integrin CD11c [9, 10], but this marker is co-expressed with CD11b on macrophages and natural killer cells [6]. Macrophage CD11c expression even seems to indicate a M1 polarization [11] which sheds new light on the possible functions of CD11c+ cells after stroke. To approach the problem of macrophage and DC identity after stroke, we used multidimensional flow cytometry to identify different members of the mononuclear phagocyte system by more specific combinations of surface markers.

## Methods

### Animals and experimental stroke

The study conformed to the Guide for the Care and Use of Laboratory Animals published by the US National Institutes of Health (NIH Publication No. 85-23, revised 1996) and was approved by local state authorities (protocol number TVV10/13). Six male mice (C57BL/6, Charles River Laboratories, Sulzfeld, Germany; 14 weeks old) were randomly assigned to a control group (naive, n = 3) and a stroke group (photothrombosis, PT, n = 3). The PT group was then replicated with three additional animals (experiment 2). Animals were singly housed with free access to food and water and kept on a 12 h/12 h light-dark cycle. PT was induced as described previously [12]. Briefly, mice were anesthetized with isoflurane (2%), injected with carprofen (5 mg/kg bodyweight s.c.) and placed in a stereotactic frame. The skull was exposed by midline incision and a cold-light photodiode was placed 2.5mm right and 1mm posterior from Bregma. Five minutes after intraperitoneal injection of Bengal rose solution (0.1mL) the brain was illuminated for 15 minutes.

### Sampling

Animals were sacrificed six days after stroke by CO2 exposure under deep isoflurane narcosis. The circulation was transcardially perfused with 100 mL Hank's buffered salt solution (HBSS, Sigma, Taufkirchen, Germany) before brains were removed. Single brain hemispheres were manually and enzymatically (Liberase TL, 2 U/mL, Roche, Mannheim, Germany) homogenized in HBSS with calcium/magnesium. Brain leukocytes were separated by Percoll solution (25%, GE Healthcare, Munich, Germany) using density gradient centrifugation as previously reported [13].

### Flow cytometry

**Figure 1:**
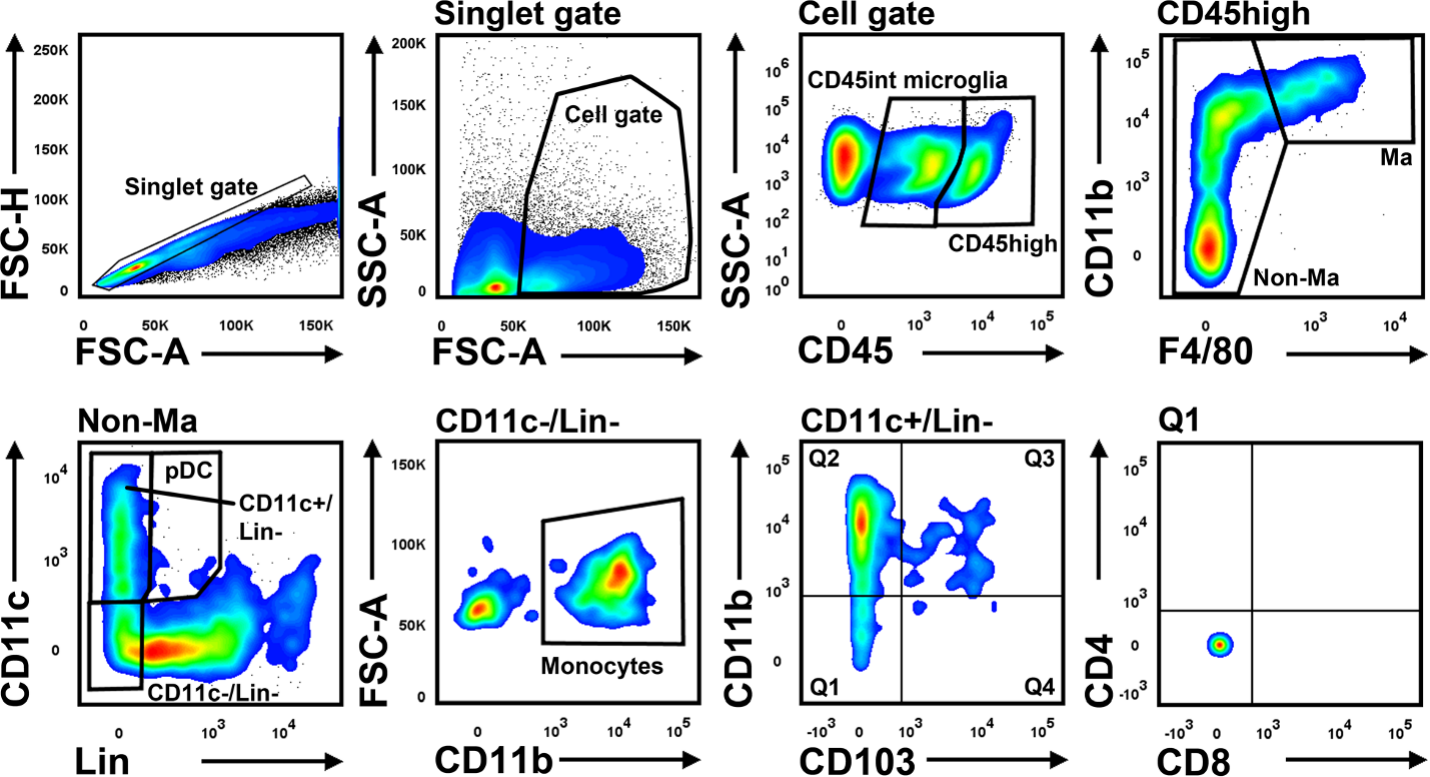
**Gating strategy for the analysis of brain leukocytes.** Lineage (Lin) antibodies include CD3 (T cells), B220 (B cells, pDC), Ly6G (neutrophils) and CD49b (NK cells, NKT cells). FSC-H, forward scatter height; FSC-A, forward scatter area; SSC-A, side scatter area; Ma, macrophages.

Cell suspensions were first incubated with an anti-mouse CD16/CD32 Fc-receptor blocking reagent (eBioscience, Frankfurt, Germany) for 10min before being labeled with the following anti-mouse antibodies: F4/80-Alexa Fluor488 (AbD Serotec Kidlington, UK), CD4-PerCP, MHCII (I-A/I-E)-PerCP, CD11b-APC, CD45.2-APC-eFluor780 (all eBioscience), CD11c-PE-Cy7, CD103-Pacific Blue (both Biolegend, San Diego, USA), CD8-Horizon V500 (BD Biosciences, Heidelberg, Germany) and biotinylated anti-lineage antibodies (CD3, B220, Ly6G, CD49b, all ebioscience) followed by incubation with phycoerythrin-labeled streptavidin (BD Biosciences). Flow cytometric analyses were performed by an investigator blinded to the group allocation using a 3-laser FACSCanto II (BD Biosciences) and analyzed by FlowJo software (Tree Star, Ashland, USA). The gating strategy is displayed in Figure 1. Total brain leukocyte counts were determined by Trucount Tube measurement (BD Biosciences) of CD45-labeled brain cell suspensions.

### Statistics

Data were presented as mean ± standard deviation. Statistical differences were determined by one-way ANOVA followed by Bonferroni’s post hoc test. P-values of 0.05 or less were considered statistically significant. Statistical analyses were performed by GraphPad Prism (Version 5.03, La Jolla, USA).

## Results

**Figure 2:**
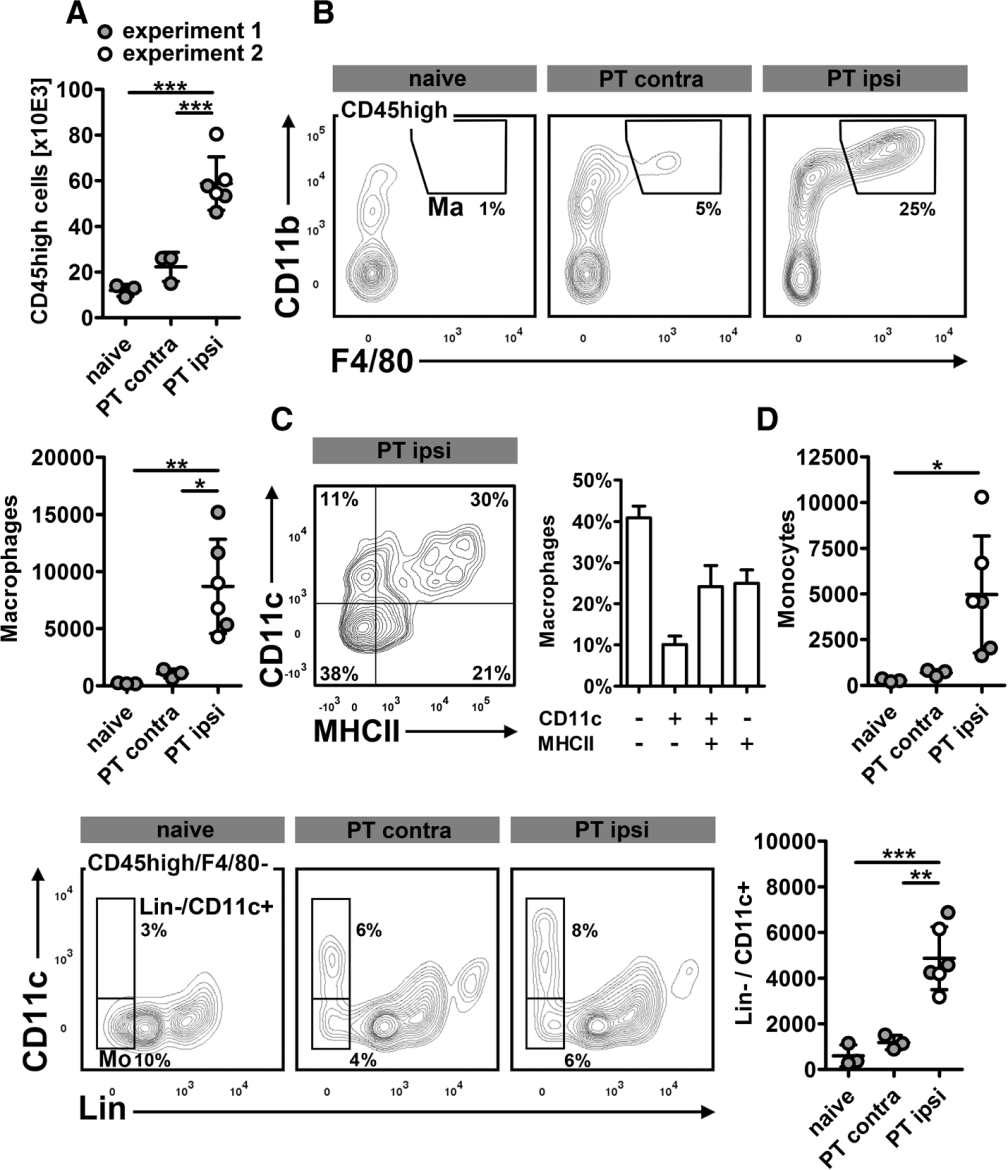
**Differentiation of mononuclear phagocytes after photothrombotic stroke (PT) in mice. (A)**, in both experiments, PT consistently caused a significant increase of CD45high + leukocytes in the ipsilateral (ipsi) hemisphere. **(B)**, subset analysis revealed a strong increase of F4/80+ macrophages which could be further categorized by CD11c and MHCII expression **(C**; experiment 2, n = 3). **(D)**, monocytes (Mo) and Lin-/CD11c+ cDC also significantly contribute to the inflammatory response to stroke. *p < 0.05, ** p < 0.01, ***p < 0.001 by one way ANOVA and Bonferronis’ post-hoc test. Contra, contralateral.

Brain infiltrating cells were defined as CD45high+ cells that could be unambiguously differentiated from CD45 intermediate (int) microglia (Figure 1, cell gate). Macrophages were delineated as CD11b+/F4/80+ cells. The remaining cells were then distinguished by a lineage (Lin) combination (CD3, B220, Ly6G, CD49b) and CD11c. Lin-/CD11c-/CD11b+ cells were categorized as monocytes, Lin-/CD11c+ as conventional DC (cDC) and Lin+ (B220+)/CD11c+ cells as plasmacytoid DC (pDC; Figure 1, non-macrophage gate).Flow cytometric quantification revealed a significant increase of CD45+ cells in the ischemic hemisphere at day 6 after stroke (naive: 115.2 ± 27.5 × 10E3 cells and PT contralateral: 107.8 ± 26.1 x 10E3 cells versus PT ipsilateral: 183.2 ± 17.1 × 10E3 cells; p < 0.01). This difference was primarily caused by CD45high leukocytes (Figure 2A) and, to a lesser degree, by CD45int microglia (naive: 103.3 ± 28.9 × 10E3 cells and PT contralateral: 85.3 ± 20.6 × 10E3 cells versus PT ipsilateral: 124.5 ± 16.7 × 10E3 cells; p > 0.05). Compared to naive controls, experimental stroke induced a 40 fold increase of F4/80+ macrophages within the ipsilateral hemisphere (Figure 2B). The majority of these cells did not co-express CD11c or major histocompatibility complex (MHC)-II, but we also observed CD11c- or MHCII-single positive and CD11c/MHCII-double positive macrophages (Figure 2C).We found a comparable increase of monocytes and Lin-/CD11c+ cDC (Figure 2D) within the ischemic hemisphere. Finally, stroke induced a slight, but statistically significant increase of pDC (naive: 135 ± 104 cells and PT contralateral: 186 ± 105 cells versus PT ipsilateral: 524 ± 215 cells; p < 0.05)

**Figure 3:**
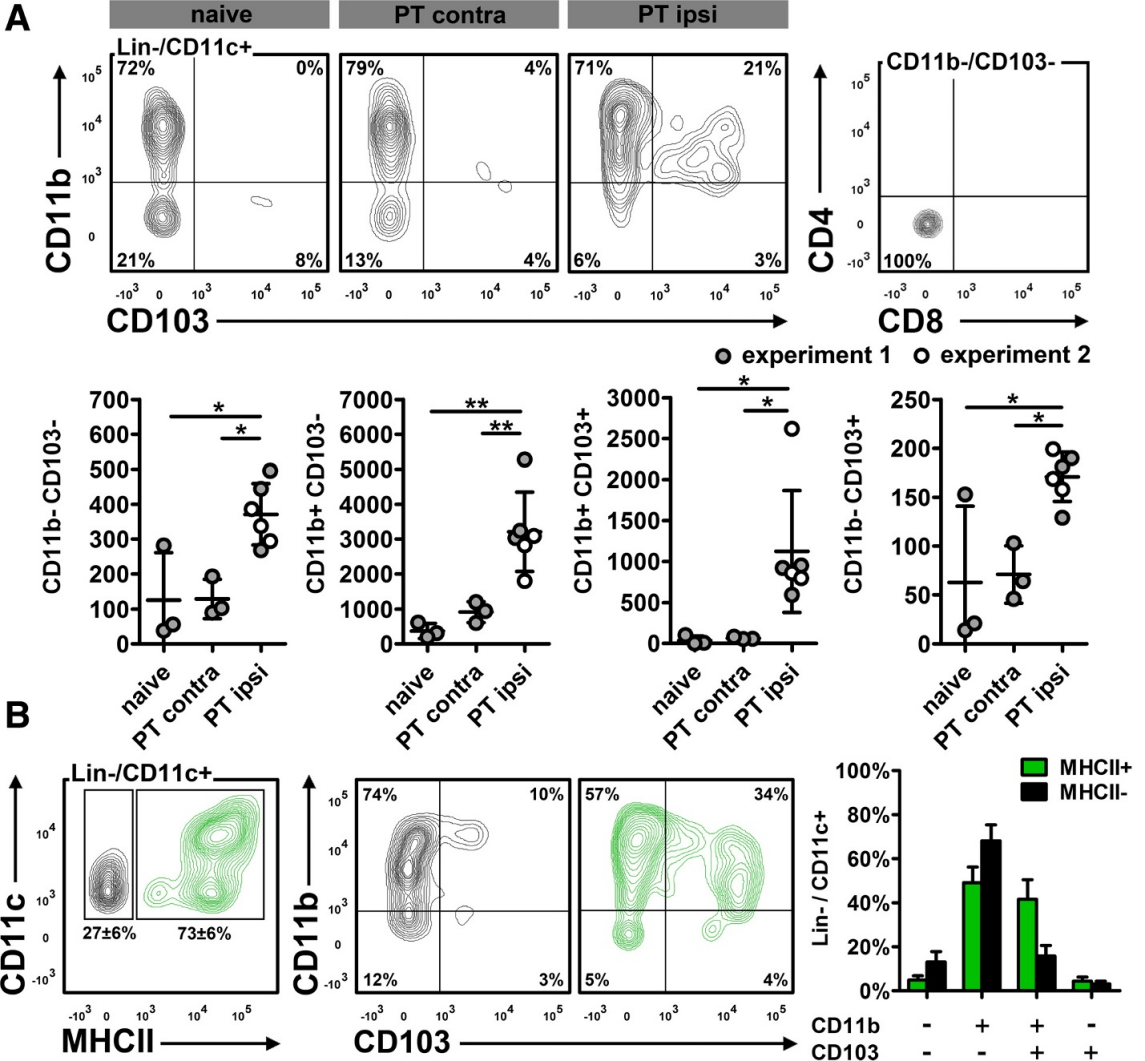
**Subdifferentiation of Lin-/CD11c+ cells. (A)**, in the ipsilateral (ipsi) hemisphere, Lin-/CD11c+ cells were primarily composed of CD11b+/CD103- and CD11b+/CD103+ cells. We found only few CD11b-/CD103+ and CD11b-/CD103- cells, but no CD4+ or CD8+ expression. **(B)**, Lin-/CD11c+ cells were predominantly MHCII+. CD11b+/CD103+ DC were enriched in the MHCII+ population (green), but also present among MHCII- cells (black; experiment 2, n = 3). *p < 0.05, **p < 0.01 by one way ANOVA and Bonferronis’ post-hoc test. Contra, contralateral.

Next, we differentiated Lin-/CD11c+ cDC by CD11b and CD103 as classical markers for non-lymphoid tissue DCs. Two major subpopulations responded to ischemic stroke: a CD11b+/CD103- population and CD11b+/CD103+ cells (Figure 3A) that may resemble migratory and tolerogenic DCs in the intestinal lamina propria [14]. CD11b+/CD103+ cells were virtually absent in naive brain tissue, but 30fold increased in the ischemic hemisphere. Neither CD11b-/CD103- cells (Figure 3A) nor CD11b+/CD103- and CD11b-/CD103+ cells (not shown) expressed CD4 or CD8.

Approximately 75% of the Lin-/CD11c+ cells were also positive for MHCII and could hence be categorized as matured cDCs (Figure 3B). However, almost 20% of the MHCII- cells were also CD11b+/CD103+, indicating that at least a part of Lin-/CD11c+/MHCII- are DCs in pre-mature state [15].

## Discussion

After stroke, the immune system is challenged by a massive release of brain antigens that are ultimately present in T cell zones of cervical lymph nodes [8]. It is likely that subsets of the mononuclear phagocyte system enter the ischemic lesion, transport antigens to draining lymph nodes and orchestrate the induction of immunity and tolerance. To understand these processes, it is necessary to dissect subsets, and eventually time course and fate of DC after stroke.

The integrin CD11c is frequently used as bona fide marker to study DC biology, but CD11c expression is described on various leukocyte subsets including macrophages, granulocytes and NK cells [6]. In this study, almost one-third of F4/80+ macrophages in the ischemic hemisphere also expressed CD11c, possibly indicating an M1-like pro-inflammatory phenotype [11]. The exclusive use of CD11c is therefore not accurate to distinguish macrophages and DC during post-stroke inflammation. The same applies for MHCII that is increasingly expressed during DC maturation [15], but also by activated macrophages [6].

There is insufficient data on non-lymphoid tissue DC phenotypes under inflammatory conditions such as stroke, but DC in tissues with physiological continuous antigen exposure such as the lung or the gut may serve as a model. Murine lung and intestinal macrophages express high levels of F4/80 and varying levels of CD11c, whereas CD11c+ DC are predominantly F4/80- [14, 16, 17]. We therefore decided to apply a two-step gating strategy to first separate macrophages by F4/80 expression before differentiating monocytes by Lin-/F4/80-/CD11c-/CD11b+ and cDC by Lin-/F4/80-/CD11c+. This approach is limited by the fact that infiltrating Ly6Clow resident monocytes may also express CD11c [18] and could finally be false positively quantified as CD11b+/CD103- cDC. However, Co-staining with MHCII revealed that the majority of Lin-/CD11c+ cells are in fact mature DCs, whereas the remaining MHCII- population may comprise of both, Ly6low resident monocytes and Ly6Chigh monocytes that are differentiating and maturing towards DC [19].

Six days after stroke, we found monocytes, macrophages, cDC and, at a lower level, pDC significantly increased in the ischemic hemisphere. The increase of cDC was primarily driven by CD11b+/CD103- cells which are derived from inflammatory monocytes and produce pro-inflammatory mediators such as IL-12, IL-23 and TNF in the inflamed colon [16]. By contrast, CD11b-/CD103+ cDC, that rather emerge from circulating DC precursors [20, 21] were barely altered in the ischemic hemisphere. Finally, we found a strong increase of CD11b+/CD103+ cDC in the ischemic hemisphere, a subpopulation that is scarcely present in naive brain tissue. These cells may resemble CD11b+/CD103+ cDC in the intestines that express high levels of CCR7, carry antigens toward draining lymph nodes and promote tolerance to ingested antigens [14]. After stroke, emigrating CD11b+/CD103+ cDC may play a pivotal role in the control of clinically relevant autoreactive immune responses to brain antigens [3].

To our knowledge, this is the first report on dendritic cell subsets during post-stroke inflammation. Some overlap may exist for Ly6Clow resident monocytes and maturating MHCII- DC, but only the detection of determining transcription factors might help to overcome these uncertainties [22]. Finally, we could identify a CD11b+/CD103+ DC subset that is well-studied in the mucosal immunity and may also adopt important functions after stroke.
